# Telephone-based goal management training for adults with mild traumatic brain injury: study protocol for a randomized controlled trial

**DOI:** 10.1186/s13063-015-0775-1

**Published:** 2015-06-02

**Authors:** Kristin R Archer, Rogelio A Coronado, Lori R Haislip, Christine M Abraham, Susan W Vanston, Anthony E Lazaro, James C Jackson, E Wesley Ely, Oscar D Guillamondegui, William T Obremskey

**Affiliations:** Department of Orthopaedic Surgery, Vanderbilt University School of Medicine, 1215 21st Avenue South, Medical Center East, South Tower, Nashville, TN 37232 USA; Department of Physical Medicine and Rehabilitation, Vanderbilt University School of Medicine, 2201 Children’s Way, Suite 1318, Nashville, TN 37212 USA; School of Medicine, Meharry Medical College, 1005 Dr. D.B. Todd Jr. Boulevard, Nashville, TN 37208 USA; Division of Allergy, Pulmonary, and Critical Care, Department of Medicine, Center for Health Services Research, Vanderbilt University School of Medicine, 1215 21st Avenue South, Medical Center East, North Tower, Nashville, TN 37232 USA; Department of Psychiatry, Vanderbilt University School of Medicine, 1601 23rd Avenue, Nashville, TN 37212 USA; Geriatric Research, Veteran’s Affairs Tennessee Valley Geriatric Research Education and Clinical Center (GRECC), Tennessee Valley Healthcare System, 1310 24th Avenue South, Nashville, TN 37212 USA; Division of Trauma and Surgical Critical Care, Department of Medicine, Vanderbilt University School of Medicine, 1215 21st Avenue South, 404 MAB 1750, Nashville, TN 37232 USA

**Keywords:** Randomized trial, Traumatic brain injury, Cognitive rehabilitation, Goal management training, Mindfulness

## Abstract

**Background:**

Approximately 1 million individuals experience a mild traumatic brain injury (TBI) and cost the United States nearly $17 billion each year. Many trauma survivors with mild TBI have debilitating and long-term physical, emotional, and cognitive impairments that are unrecognized at trauma centers. Early intervention studies are needed to address these impairments, especially cognitive deficits in executive functioning. Goal management training (GMT) is a structured cognitive rehabilitation program that has been found to improve executive functioning in patients with moderate to severe TBI. The current study adapted the GMT program for telephone delivery in order to improve the accessibility of rehabilitation services in a patient population with multiple barriers to care and significant yet unrecognized cognitive impairment. The primary objective of this study is to examine the efficacy of telephone-based GMT for improving executive functioning, functional status, and psychological health in trauma survivors with mild TBI.

**Methods/design:**

This study is a three-group randomized controlled trial being conducted at a Level I trauma center. Ninety trauma survivors with mild TBI and cognitive deficits in executive functioning will be randomized to receive telephone-based GMT, telephone-based education, or usual care. GMT and education programs will be delivered by a physical therapist. The first in-person session is 1 h and the remaining six telephone sessions are 30 min. A battery of well-established cognitive tests will be conducted and validated questionnaires will be collected that measure executive functioning, functional status, and depressive and posttraumatic stress disorder symptoms at 6 weeks, 4 months, and 7 months following hospital discharge.

**Discussion:**

This study supports a telephone-delivery approach to rehabilitation services in order to broaden the availability of evidence-based cognitive strategies.

**Trial registration:**

This trial was registered with Clinicaltrials.gov on 10 October 2012, registration number: NCT01714531.

## Background

Approximately 2.5 million individuals are hospitalized each year due to traumatic injuries [1, 2], with over half experiencing a brain injury. Patients with moderate to severe traumatic brain injury (TBI) have evident debilitating cognitive and functional impairments. However, mild TBI is a silent epidemic that can result in long-term or permanent impairment and disability that is under-managed at trauma centers [3, 4]. The Centers for Disease Control (CDC) estimates that more than 1 million individuals experience a mild TBI and cost the United States (U.S.) nearly $17 billion each year [4].

There are considerable symptoms as a consequence of mild TBI including poor concentration, lethargy, confusion, disorientation, and irritability [5]. Physical, emotional, and cognitive deficits as a result of these symptoms can become chronic and disabling leading to vocational and social disabilities [5]. Physical symptoms such as impaired gait, persistent headaches, fatigue, and dizziness may continue for several months up to many years, delaying one’s ability to return to work [6, 7]. Depressive and posttraumatic stress disorder (PTSD) symptoms are extremely common in individuals with cognitive impairment [8, 9], with mild TBI being the triggering event for an episode of depression in some individuals [10]. Thirty to forty percent of trauma survivors with mild TBI have depressive symptoms and 20 % to 30 % have PTSD within the first year of recovery. Long-term cognitive consequences of mild TBI include deficits in attention, memory, and most importantly, executive functioning [11, 12, 7].

Executive functions are those involved in complex cognitions such as planning, initiating activities, and monitoring and inhibiting, which enable individuals to engage in purposeful, goal-directed behaviors (for example, balancing a checkbook and understanding social cues) [13, 14]. Deficits in executive functioning are the most disabling of all cognitive impairments and affect a person’s ability to manage effectively in one’s personal and professional life. Current literature demonstrates that deficits in executive functioning contribute to reduced quality of life, difficulty in returning to work, and persistent psychological distress in patients following head injury [15, 16]. Deficits in executive functioning may also contribute to the development and maintenance of depression and PTSD [17], with studies suggesting that cognitive impairment and psychological distress share neuroanatomic and pathophysiologic correlates [17, 18].

Current literature supports the effectiveness of cognitive rehabilitation for improving cognitive, functional, and psychological health in patients with identified brain injury [19, 20]. Cognitive rehabilitation retrains previously learned skills, increases awareness and acceptance of cognitive impairments, and teaches self-confidence and self-efficacy for coping with emotional distress. Data show that cognitive interventions are effective in a variety of settings (for example, inpatient, outpatient, and home) and when delivered by various professionals in different disciplines [21, 20]. Cognitive rehabilitation has not been traditionally offered or studied in patients with mild TBI. This population of trauma survivors has limited access to care due to underdiagnosis, as well as financial constraints and mobility issues that typically render clinic-based rehabilitation impractical.

Goal management training (GMT) is a structured form of cognitive rehabilitation that has been found to improve executive functioning in patients with moderate to severe brain injury and older adults with cognitive impairment [22, 23]. GMT uses metacognitive strategies to improve patients’ ability to organize and achieve goals in ‘real-life’ situations. GMT participants are taught to be reflective (that is, to ‘stop and think’) prior to making decisions and executing specific tasks, and to achieve success by dividing tasks into manageable units, so as to increase the likelihood that these tasks are completed.

The purpose of this study is to examine the efficacy of a telephone-based GMT program for improving executive functioning, functional status, and depressive and PTSD symptoms in trauma survivors with mild TBI. The GMT program will be compared to a telephone-based education program and usual care at 4 months (treatment completion) and 7 months following hospital discharge. Emerging research suggests that telephone rehabilitation may be a feasible and effective alternative (with much broader applicability) to clinic-based interventions [24–27]. Researchers have also suggested that rehabilitation conducted in a patient’s well-known and natural environment may facilitate and enhance the transfer of skills to the everyday living setting [28].

## Methods/design

### Study design

**Figure 1:**
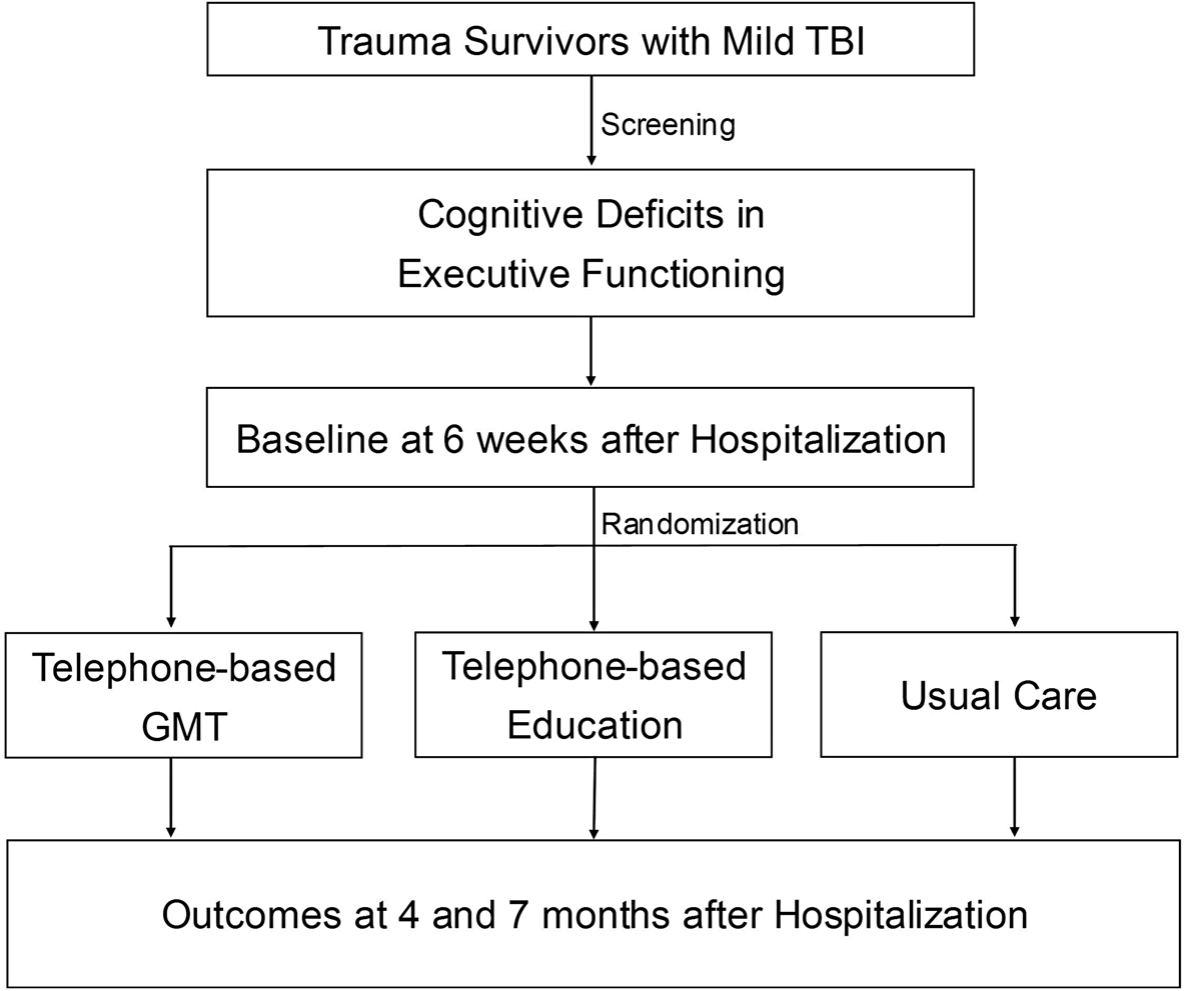
Study flow diagram

This study is a three-group randomized controlled trial conducted at a Level I trauma center. Figure 1 depicts the overall study design with assessments at 6 weeks (baseline) and 4 and 7 months after hospital discharge (see ClinicalTrials.gov and NCT01714531 for more information). The investigators, participating surgeons, research personnel conducting the assessments, and patients will be blinded to group assignment. Potential subjects will be informed that they will be randomly assigned to one of two different educational treatments or usual care. Participants will be asked not to discuss study procedures with their treating surgeon, medical staff, and research personnel.

### Study population

Ninety English-speaking adults with mild TBI and cognitive deficits in executive functioning who are admitted to a Level I trauma center will be recruited for this study. Mild TBI will be determined through a medical chart review and patient interview questions using American Congress of Rehabilitation Medicine guidelines [29]. The guidelines include at least one of the following: (1) any period of loss of consciousness; (2) any loss of memory for events immediately before or after the accident; (3) any alteration in mental state at the time of the accident; and (4) focal neurological deficit(s) that may or may not be transient; but where the severity of the injury does not exceed the following: (1) posttraumatic amnesia not greater than 24 h; (2) after 30 min, an initial Glasgow Coma Scale of 13 to 15; and (3) loss of consciousness of approximately 30 min or less. Eligible participants with mild TBI will also be screened for presence of cognitive deficits in executive functioning. Deficits are defined for this study as one standard deviation (SD) below the norm referenced mean on any one of the following neuropsychological tests: Delis-Kaplan Executive Function System (D-KEFS) Tower Test, Trail Making Test B (Trails B), and FAS test [13, 30].

### Exclusion criteria

Patients will be excluded from the study if they meet any of the following criteria: (1) documented evidence of moderate to severe TBI; (2) current alcohol or substance dependence within the last 6 months; (3) preexisting cognitive impairment as determined by a score greater than 3.3 on the short form of the Informant Questionnaire of Cognitive Decline in the Elderly [12, 31]; (4) neurological history other than TBI (for example, premorbid epilepsy, multiple sclerosis, Alzheimer’s disease); (5) history of schizophrenia, psychotic disorder, or suicidal intent; and (6) inability to provide a telephone number and a stable address.

### Procedures

Written informed consent will be obtained from all study participants prior to study enrollment. Participants will be screened for mild TBI, preexisting cognitive impairment, alcohol and substance dependence, and cognitive deficits in executive functioning. Those that pass the screening phase will be asked to complete a baseline assessment (6 weeks after hospital discharge) and follow-up assessments at 4 and 7 months following hospitalization. Table 1 summarizes the data collection procedures across the baseline and follow-up time points. Assessments will consist of cognitive tests and questionnaires that measure cognitive functioning, functional status, and depressive and PTSD symptoms. Patients will also be asked to answer demographic and health questions at the baseline assessment. Clinical characteristics will be extracted from the medical record. All data will be entered into the Research Electronic Data Capture system (REDCap, a secure, web-based application designed exclusively to support data capture) [32].

**Table 1:** Data collection schedule after hospital discharge

	6 weeks	4 months	7 months
Patient characteristics			
Age, gender	x		
Race/ethnicity	x		
Marital status	x		
Educational level	x		
Insurance status	x		
Height/weight	x		
Smoking status	x		
Working status	x		
Comorbid conditions	x		
Recovery expectations	x		
Clinical characteristics			
Glasgow Coma Scale	x		
Injury Severity Score	x		
Mechanism of injury	x		
Type of injury	x		
Surgical procedure	x		
Length of hospital stay	x		
Intensive care unit stay	x		
Ventilator days	x	x	x
Medications	x	x	x
Complications	x	x	x
Executive functioning			
D-KEFS Tower Test	x	x	x
Trails B	x	x	x
FAS test	x	x	x
SART	x	x	x
Hotel Task	x	x	x
Dysexecutive Questionnaire	x	X	x
Cognitive Failures Questionnaire	x	x	X
Functional status			
Functional Activities Questionnaire	x	x	x
Quality of Life After Brain Injury	x	x	x
Psychological			
Patient Health Questionnaire-9	x	x	x
PTSD Checklist - Civilian Version	x	x	x

*D-KEFS* Delis-Kaplan Executive Function System, *SART* Sustained Attention to Response Test, *PTSD* posttraumatic stress disorder

### Randomization

Participants will be randomized to one of the three groups (telephone-based GMT, telephone-based education, usual care) in a 1:1:1 ratio. Block size will be determined randomly with the patient as the unit of randomization. A randomization list will be computer generated and administered thought the REDCap system. Randomization will occur immediately after the baseline assessment at 6 weeks. Surgeons and research personnel conducting the assessments will be unaware of group assignment.

### Interventions

#### Telephone-based GMT

**Figure 2:**
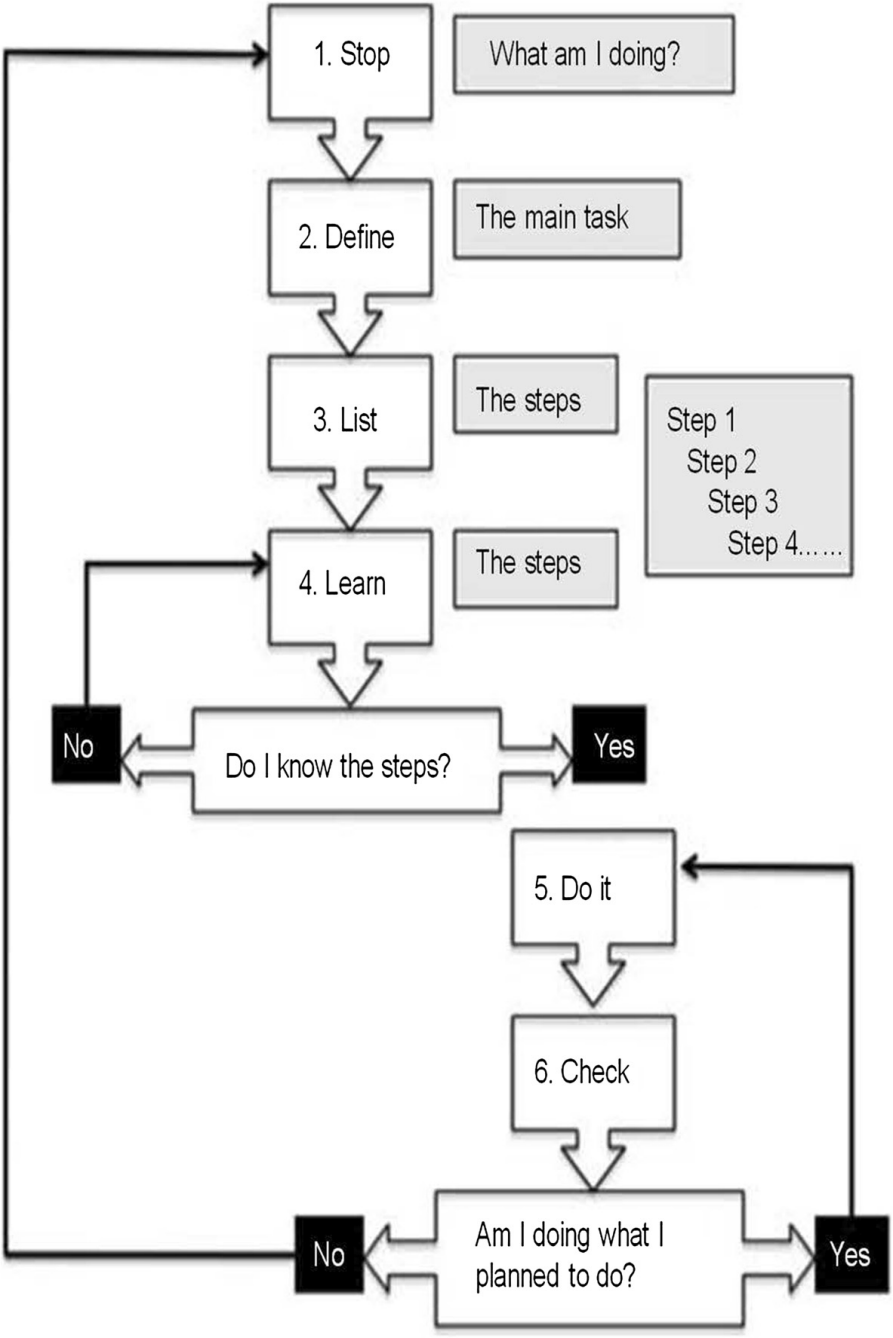
Goal management training overview

The telephone-based GMT program will include seven sessions delivered by a physical therapist (Table 2). GMT was originally conceptualized by Robertson [33] and derived from Duncan’s [34] theory of goal neglect. Levine and colleagues expanded on GMT and tested a standardized protocol and treatment manual in patients with moderate to severe brain injury and in older adults with cognitive impairment [23, 35, 36, 21]. The GMT intervention targets cognitive deficits in executive functioning that impact a person’s ability to carry out daily tasks. This current study has adapted the GMT intervention to include mindfulness techniques [37] and to be delivered over the telephone, in collaboration with Dr. Brian Levine. The first session is 1 h and conducted in-person to provide participants with a session-by-session treatment manual. The remaining six sessions are 30 min and are conducted once a week over the phone. Sessions focus on increasing awareness of one’s thoughts and experiences and increasing self-efficacy. Participants learn how to use mindful attention and goal setting to recognize and stop ‘absentmindedness’ and ‘automatic pilot’ in order to reduce daily errors and ‘slips’ (Fig. 2). Each session builds upon the content of the previous session. Weekly homework is personally tailored based on patient goals.

**Table 2:** Summary of goal management training intervention by session

Session 1: Slip-ups	Overall introduction, define goals and absentminded slips, raise awareness of consequences of slips, introduce present-mindedness and mindful practice in daily life
Session 2: Stop the automatic pilot	Define automatic pilot and how it leads to errors, learn how to ‘STOP’ automatic pilot, practice present-mindedness
Session 3: The mental blackboard	Define the mental blackboard, learn how to ‘STOP’ and check mental blackboard, staying in the present through breathing
Session 4: State your goals	Define goals, learn how to state goals, practice ‘STOP’ and ‘STATE’ and breath focus to become present-minded
Session 5: Making decisions	Define competing goals, learn how to understand emotional reaction to indecision, practice ‘STOP-STATE’ to reduce stress and indecision
Session 6: Splitting tasks into subtasks	Define overwhelming goals, learn how to split goals, practice ‘STOP-STATE-SPLIT’
Session 7: Checking (STOP)	Learn how to recognize errors in ‘STOP-STATE-SPLIT’ cycle, review how to use ‘STOP’ to monitor daily tasks, review strategies for being present-minded, wrap-up

#### Telephone-based education

The telephone-based education group will receive an educational program that is matched to the GMT intervention in terms of session length and contact with the study therapist. The education program includes seven sessions delivered by a physical therapist (Table 3). Material was developed by Levine *et al*. [23] based on material commonly employed in rehabilitation centers and has been successfully used in several studies as a comparison to the GMT intervention [35, 38]. Sessions address education on brain function and cognitive principles of memory, attention, language, perception, and motor skills. Education on stress reduction, sleep hygiene, energy management, exercise, communication, and nutrition are also provided. The first session is conducted in-person to provide participants with a session-by-session treatment manual. The remaining six sessions are conducted once a week over the phone.

**Table 3:** Summary of education intervention by session

Session 1: Introduction	Overall introduction, explain the goals of the program, define basic brain anatomy and consequences of trauma
Session 2: Brain activity	Explain importance of keeping brain active, define the assessment of brain activity
Session 3: Memory I	Explain the importance of memory, define the types of memory and memory processes
Session 4: Memory II	Review memory and the brain, learn how memory breaks down, define functional implications of memory loss
Session 5: Attention and executive function	Define attention and executive functioning, learn how attention and executive functioning breaks down
Session 6: Lifestyle I	Explain importance of stress, sleep, and exercise on brain function, define the influence of lifestyle on recovery
Session 7: Lifestyle II	Explain importance of nutrition, energy management, and communication on brain function, review healthy lifestyle for recovery, wrap-up

#### Usual care

Participants in the usual care group will receive usual care as determined by the treating surgeon. Usual care may include referral to a physical therapist, occupational therapist, psychiatrist, and/or psychologist. At the end of the intervention phase, participants will be asked whether they had, on their own initiative, followed a course to improve their cognitive functioning.

### Quality assurance

One study physical therapist will complete training in both the GMT and education programs. Formal training will occur with the principal investigator (PI) of the study (KRA) and an experienced neuropsychologist (JCJ). Written and skills competency tests will be completed at the end of training. After passing both tests (scores >85), the GMT and education treatments will be implemented with study staff and a pretest of both programs will occur with one patient. All sessions during the pretest will be audiotaped and reviewed to evaluate adherence to the treatment protocol and structured manual.

Our treatment integrity protocol includes detailed session-by-session treatment manuals for the telephone-based groups and ongoing supervision to ensure accurate and consistent treatment delivery (provided via weekly clinical team meetings). The study physical therapist’s adherence to procedures will be assessed by audio recording all sessions and randomly selecting sessions for the investigators to review using standardized fidelity checklists. The study physical therapist will also complete a checklist of all the components delivered during each session and make note of any protocol deviations. If the integrity of the treatments is compromised, the study therapist will be retrained and 100 % of audiotapes will be reviewed until problems are addressed.

### Primary outcome measures

#### Executive functioning

Executive functioning will be measured using a battery of widely used and previously validated cognitive tests and patient-reported questionnaires (Table 4).

**Table 4:** Cognitive tests and patient-reported questionnaires for assessing executive functioning

Test	Description	Executive function	Outcome	Scoring	Interpretation
DKEFs Tower Test [30]	A timed test that involves constructing towers of discs on a set of pegs with rules for movement and setup	Planning, sustained attention	Total number of moves	Age-adjusted scaled score [range: 1 to 19]	Higher scaled scores indicate better performance
					Scaled scores ≤7 indicate significant impairment
Trails B [30, 39–41]	A timed test that involves drawing a line between a series of alternating numbers and letters	Attentional control, cognitive flexibility, set shifting	Total time	Age, education, and sex-adjusted T-score	Higher T-scores indicate better performance
					T-scores ≤35 indicate significant impairment
FAS [13]	A timed test that involves generating as many words beginning with the letters F, A, and S	Verbal fluency	Total number of words	Age, education, and sex-adjusted T-score	Higher T-scores indicate better performance
					T-scores ≤35 indicate significant impairment
SART [44–47]	A timed computer test that involves a go/no-go task using single-digit numbers	Sustained attention and inhibition	Errors of commission and omission, reaction time	Sum of errors Reaction time	Higher number of errors and slower reaction time indicate poorer performance
Hotel Task [48, 49]	A timed test that involves a real-life multitasking situation with different task components	Planning, organization	Total time, time spent on each task, number of tasks attempted	Deviation time from optimal time allocation	Greater deviation time indicates poorer performance
DEX [50, 52]	A 20-item questionnaire that measures behavioral change and difficulties with executive functions	Changes in emotion, personality, motivation, behavior, and cognition	Total score	Summed score [range: 0 to 80]	Higher scores indicate greater cognitive impairment
CFQ [51, 53, 54]	A 25-item questionnaire that measures daily mental errors related to attention and cognition	General everyday life cognitive failures	Total score	Summed score [range: 0 to 100]	Higher scores indicate greater cognitive impairment Scores >38 indicate cognitive difficulties

*D-KEFS* Delis-Kaplan Executive Function System, *SART* Sustained Attention to Response Test, *DEX* Dysexecutive Questionnaire, *CFQ* Cognitive Failures Questionnaire

The D-KEFS Tower Test assesses the ability to plan and strategize efficiently and requires participants to move discs across three pegs until a tower is built using the fewest number of moves possible [30]. D-KEFS Tower Test is timed, but participants are unaware of specific time constraints. If the tower is not built within the allotted time, participants receive a score of 0. Completed D-KEFS Tower Test scores are adjusted for age and converted into a scaled score that ranges from 1 to 19, with higher scores reflecting better performance. The D-FEKS Tower Test has demonstrated moderate correlations with self-reported executive functioning and has been found to be sensitive and specific for brain lesion diagnosis [30].

Trails B is a time-based test that measures set shifting and cognitive flexibility [30]. Participants are asked to draw a line between a series of alternating numbers and letters according to a specified sequence. Trails B has acceptable test-retest reliability [39] and good convergent and predictive validity with significant associations with self-reported executive functioning and functional status in patients with TBI and older adults [40, 41]. The FAS assesses verbal (letter) fluency and is a valid and sensitivity measure of frontal lobe function [13]. Participants are given 1 min to generate as many words as they can for each of the letters F, A, and S. The FAS exhibits moderate correlation with measures of executive functioning after TBI and good sensitivity and specificity for patients with dementia [42, 43]. Trails B and FAS scores are adjusted for age, education, and gender and converted to T-scores, with a norm referenced mean of 50.

The Sustained Attention to Response Test (SART) is a go/no-go computer test that identifies failures of sustained attention [44]. Participants are instructed to respond to randomly presented single numbers (one through nine) every 1.15 s, except for a single no-go number (for example ‘three’). The number of errors (commission and omission) and reaction time are recorded and used as scores for the SART. The SART has good sensitivity at discriminating attention error rates of TBI patients [45, 46] and good convergent validity through associations with self- and informant-reported measures of everyday attention failure and lapses [46, 47].

The Hotel Task is a measure of planning and organizational ability and involves the participant modeling a real-life multitasking situation as a hotel manager [48]. The participant is asked to try and complete five different tasks: compiling bills; sorting a charity collection; looking up telephone numbers; sorting conference labels; proofreading the hotel leaflet. In order to complete all five tasks, the participants must distribute their time equally across the total 15-min allotment (that is, 3 min per task). Scoring of the Hotel Task is the total deviation from optimal time allocation. The Hotel Task is a sensitive measure for detecting frontal dysfunction in various conditions [48, 49].

Patient-reported executive functioning will be measured using the Dysexecutive Questionnaire (DEX) [50] and the Cognitive Failures Questionnaire (CFQ) [51]. The DEX is a 20-item questionnaire that assesses behavioral changes in executive functioning related to the areas of inhibition, memory, intention, and affect. Items on the DEX are scored using a 5-point Likert scale ranging from 0 (never) to 4 (very often) with total scores ranging from 0 to 80. The DEX has demonstrated good internal consistency and moderate correlations with other measures of patient-reported executive functioning in adults with dementia [52]. The CFQ is a 25-item questionnaire that assesses daily mental errors associated with distractibility, blunders, names, and memory [51]. Items on the CFQ are scored using a 5-point Likert scale ranging from 0 (never) to 4 (very often) with total scores ranging from 0 to 100. CFQ scores greater than 38 have been reported to indicate persistent cognitive difficulties [53]. The CFQ has been shown to have excellent psychometric properties and moderate to high correlations with cognitive tests and questionnaires in patients following head injury [54].

### Secondary outcome measures

#### Functional status

The Functional Activities Questionnaire (FAQ) [55] and Quality of Life after Brain Injury Overall Scale (QOLIBRI-OS) [56] will be used to assess functional status. The FAQ is a 10-item questionnaire measuring a person’s ability to perform daily tasks such as writing checks, shopping, preparing meals, and others [13, 55]. Items on the FAQ are scored using a 4-point Likert scale ranging from 0 (normal) to 3 (dependent). The FAQ has excellent inter-rater reliability and is highly correlated with other instrumental activities of daily living (IADL) measures such as the Lawton and Brody’s IADL [55]. The QOLIBRI-OS is a brief 6-item measure that assesses overall satisfaction with physical condition, cognition, emotions, function, personal/social life, and current situation/future prospects in people with TBI [56]. Items on the QOLIBRI-OS are scored using a 5-point Likert ranging from 1 (not at all) to 5 (very). Scores are summed and converted to a percentage where 0 % represents the lowest and 100 % the highest possible health-related quality of life. The QOLIBRI-OS is a unidimensional scale that demonstrates good reliability and correlates highly with the full 37-item QOLIBRI scale and other measures of health-related function [56].

#### Psychological health

The 9-item Patient Health Questionnaire (PHQ-9) will assess depressive symptoms with items scored using a 4-point Likert scale from 0 (not at all) to 3 (nearly every day) [57]. Total scores on the PHQ-9 can range from 0 to 27. Scores of 10 or greater are commonly used cutoff points for clinically significant depressive symptoms [58]. In a psychometric study of the PHQ-9 in persons with TBI, the instrument demonstrated acceptable test-retest reliability and is a sensitive and specific measure when compared to a diagnosis of major depression [59]. The PTSD Checklist-Civilian Version (PCL-C) is a 17-item questionnaire that will be used to measure PTSD symptoms [60]. Patients rate questions about how much they are bothered by particular symptoms during the past month using a 5-point Likert scale from 1 (not at all) to 5 (extremely). The PCL-C has demonstrated acceptable test-retest reliability and internal consistency values, and good convergent validity with moderate to high correlations with other PTSD instruments and measures of anxiety and depression in patients with traumatic injury [61]. Studies have also found that trauma survivors with PCL-C scores equal to or greater than 45 have a 75 % probability of developing symptoms consistent with a diagnosis of PTSD [62, 63].

### Sample size

We estimated power based on a target of 90 participants (30 per group) with complete follow-up data on 72 (85 %) by the 7-month follow-up. Power was estimated by generating simulated data, and then using simulated data to try and estimate the original model parameters. Simulated datasets were generated from available pilot data. Control subjects were resampled from control individuals in the pilot data, and treatment subjects were also resampled from control individuals, but with the target effect size added to the sampled values. Power was estimated by fitting Bayesian models to each of the simulated datasets for each response variable and recording the proportion of calculated 95 % credible intervals for effect sizes that excluded zero. There will be sufficient power to detect the following effect sizes: 2.0 points on the D-KEFS Tower test, 10.0 points on the Trails B and FAS tests, 4.0 and 6.0 points on the DEX and CFQ instruments, respectively, and minimum detectable differences of 23 % for depressive symptoms and 19 % for PTSD symptoms.

### Data analysis

All data will be explored numerically and graphically for normality and appropriateness of parametric statistical testing. Analyses will be conducted using models with either original data or suitably transformed data (for example, log-linear transformation) or nonparametric analyses if necessary. Baseline variables will be summarized using appropriate descriptive statistics and compared across groups. The characteristics of the patients who are lost to follow-up will be compared to those who complete the follow-up assessments. For each outcome, we will perform longitudinal mixed-effects regression analyses, with a random intercept for patient to account for the correlation among observations from the same patient. We will examine possible nonlinear effects of the treatment over time. A random slope over time may be included to allow a separate slope to be estimated for each patient. We will fit the model with an independent conditional covariance structure and an autoregressive structure and choose the best data-supported model based on the deviance information criteria or a related criterion. The primary analysis will be intent-to-treat; missing observations due to dropout and other reasons not related to the treatments will be handled with multiple imputation methodology [64]. Statistical significance will be *P* <0.05. All analyses and reporting will be consistent with Consolidated Standards of Reporting Trials (CONSORT) guidelines. The data analysis plan will be fully specified and approved prior to completion of data collection.

### Ethics

Ethical approval has been received from Vanderbilt Institutional Review Board (IRB# 111484) at the participating center and prospectively registered at www.ClinicalTrials.gov (NCT01714531).

## Discussion

The proposed study will focus on a patient population that has significant yet clinically unrecognized and unmanaged cognitive impairment in the vital domain of executive functioning. Assessment and treatment of cognitive impairment in trauma survivors at Level I trauma centers is currently limited to patients with moderate to severe TBI. We propose to identify patients with mild TBI and clinically significant impairment in executive functioning and implement a targeted evidence-based cognitive rehabilitation program. Since previous investigations have suggested that deficits in executive functioning may contribute to the development and maintenance of depression and PTSD [17, 18], our intervention also has the potential to ameliorate depressive and PTSD symptoms during the first year of recovery following major trauma. Innovative rehabilitation interventions such as our GMT program have the potential to address poor return to work rates and profound functional and psychological disability noted in trauma survivors with mild TBI.

This study will have a direct impact on traditional rehabilitation practice. Our interventional approach broadens the availability of evidence-based cognitive strategies by expanding implementation from traditional providers, such as occupational therapists, speech-language pathologists, and neuropsychologists, to physical therapists. Compelling data are needed to support the expanding role of the physical therapist in integrating cognitive and functional strategies into patient management. This is especially important since trauma survivors are commonly referred to physical therapists during the early recovery period to address physical impairments and disability. Physical therapists are in a unique position to assess and manage both the physical and cognitive consequences of injury.

Our cognitive rehabilitation intervention will also serve to accelerate a telephone-delivery approach to rehabilitation services. Teletherapy has been used effectively in adults with chronic medical conditions and depression [65–68]. In patients with brain injury, Salazar and colleagues [69] found no significant differences in outcomes between in-hospital and telephone-based cognitive rehabilitation in military personnel with moderate to severe closed head injury. Additional research is needed to overcome common perceptions that visual contact is necessary for effective treatment. Telephone-based rehabilitation appears to be a promising approach to service delivery in patients with cognitive deficits and multiple barriers to effective treatment (that is, insurance and transportation limitations, work instability, and lack of social support and community resources). The proposed study extends the telephone-delivery model in order to improve the accessibility of effective cognitive strategies for trauma survivors.

We anticipate several difficulties in implementing the study protocol. First, the cognitive tests are time intensive and require in-person visits, which may negatively affect patient enrollment and retention. Second, we anticipate patients having a lack of awareness regarding cognitive deficits. This diminished understanding of the need for cognitive rehabilitation may impact enrollment as well as engagement in the study programs. Third, we also anticipate that completing the in-person screening and baseline assessment during the first 6 weeks following hospital discharge may be difficult due to high levels of opioid use, moderate to severe pain levels, injury to the hand or arm, and financial and geographic constraints. However, we were interested in testing our interventional approach during the early postoperative period. The National Academy of Sciences Committee on Cognitive Rehabilitation Therapy for Traumatic Brain Injury recommends that further research is needed to test the efficacy of cognitive rehabilitation therapy in individuals with milder injuries and during the subacute phase [20].

A limitation of the design of this study includes the 7-month follow-up, which impacts the ability to assess sustainability of study results. However, the priority was having adequate statistical power to detect efficacy rather than longitudinal follow-up. Serial neuropsychological assessments can result in practice effects and this will be addressed methodologically using the Reliable Change Index [13]. A potential limitation of a longitudinal study in trauma survivors is that intervening events could affect outcomes. Therefore, an intervening events questionnaire will be used to track rehospitalization, additional surgery, complications, and new or continuing use of opioid or psychoactive medications. We will use these data to control for effects of intervening events on outcomes across groups. Finally, dose–response is an important issue for the proposed study. Secondary analyses to examine the number of sessions completed will begin to explore the dose–response relationship. A next step will be to conduct a multicenter trial to further validate the telephone-based GMT intervention and improve generalizability of findings.

This study will be the first to investigate systematically a physical therapist-delivered, telephone-based cognitive rehabilitation program in patients with mild head injuries. Innovative rehabilitation interventions and delivery methods are needed to improve outcomes in trauma survivors with significant yet unrecognized cognitive impairment. Early interventional studies are also needed to address the moderate to severe cognitive, physical, and emotional impairments associated with mild TBI, especially cognitive deficits in executive functioning. There are currently no standards of treatment and early assessment and management of mild TBI are critical for optimal recovery. Overall, this line of work has the potential to benefit a large population of trauma survivors by enhancing their ability to return to a productive life both inside and outside the home.

## Trial status

Recruitment was completed in February 2015. This study is currently in the follow-up phase.

## Abbreviations

CDC: Centers for Disease Control
CFQ: Cognitive Failures Questionnaire
DEX: Dysexecutive Questionnaire
D-KEFS: Delis-Kaplan Executive Function System
FAQ: Functional Activities Questionnaire
GMT: Goal Management Training
IADL: instrumental activities of daily living
NIDDR: National Institute on Disability and Rehabilitation Research
PI: principal investigator
PCL-C: PTSD Checklist-Civilian Version
PHQ-9: Patient Health Questionnaire
PTSD: posttraumatic stress disorder
QOLIBRI-OS: Quality of Life after Brain Injury Overall Scale
SART: Sustained Attention to Response Test
SD: standard deviation
TBI: traumatic brain injury
